# Cardiovascular Complications in Hematopoietic Stem Cell Transplanted Patients

**DOI:** 10.3390/jpm12111797

**Published:** 2022-10-31

**Authors:** Ying Zhao, Rui He, Sandra Oerther, Weiying Zhou, Massoud Vosough, Moustapha Hassan

**Affiliations:** 1 Experimental Cancer Medicine, Department of Laboratory Medicine, Karolinska Institute, 14186 Huddinge, Sweden; 2 Clinical Research Center and Center of Allogeneic Stem Cell Transplantation (CAST), Karolinska University Hospital Huddinge, 14186 Stockholm, Sweden; 3 Department of Pharmacology, College of Pharmacy, Chongqing Medical University and Chongqing Key Laboratory of Drug Metabolism, Chongqing 400016, China; 4 Department of Regenerative Medicine, Cell Science Research Center, Royan Institute for Stem Cell Biology, ACECR, Tehran 1999614414, Iran

**Keywords:** hematopoietic stem cell transplantation, cardiovascular complications, anthracyclines, cyclophosphamide, graft-versus-host disease, echocardiography, congestive heart failure (CHF), left ventricular ejection fraction (LVEF)

## Abstract

Hematopoietic stem cell transplantation (HSCT) is the only curative treatment for many patients suffering from hematologic malignancies, solid tumors, inborn errors of metabolism or genetic disorders. Despite decades of successful HSCT, clinical outcomes are still far from satisfactory due to treatment-related complications, including graft-versus-host disease (GvHD) and cardiovascular complications (CVC). CVC may affect patients in the acute period post-HSCT; however, the occurrence is far higher among long-term survivors. Induction treatment using cardiotoxic treatments, e.g., anthracyclines and radiotherapy, conditioning regimens containing cyclophosphamide, and post-HSCT comorbidities, including GvHD, are factors contributing to CVC. Cardiac function evaluation prior to and post-transplantation is an important strategy for choosing the proper conditioning regimen, HSCT protocol and post-HSCT supportive care. Cardiac systolic function evaluation by echocardiography, in addition to serum cardiac biomarkers, such as troponins and brain natriuretic peptides, is recommended as a routine follow-up for HSCT patients. Angiotensin-converting enzyme inhibitors, angiotensin-II-receptor blockers, and beta-blockers, which are mostly used for the treatment of chemotherapy-induced cardiotoxicity, might be used as treatments for HSCT-related CVC. In summary, the present review reveals the urgent need for further investigations concerning HSCT-related CVC both at the preclinical and clinical levels due to the lack of knowledge about CVC and its underlying mechanisms.

## 1. Introduction

**Figure 1:**
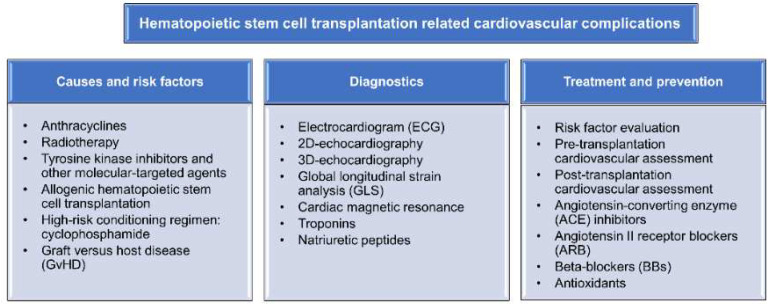
Schematic overview of hematopoietic stem cell transplantation-related cardiovascular complications.

Hematopoietic stem cell transplantation (HSCT), also called bone marrow transplantation (BMT), is a curative and probably the only treatment for many patients with advanced and high-risk hematological malignancies, e.g., leukemia, lymphoma, myelodysplastic syndrome (MDS), and myeloma, when chemotherapy and radiation have become insufficient to cure the disease. Furthermore, several studies have shown that HSCT is the only treatment for bone marrow failure syndromes, immune deficiencies, metabolic and genetic disorders such as Hurler syndrome, Hunter syndrome, severe combined immune deficiency (SCID), Wiskott syndrome, aplastic anemia, sickle cell anemia and Gaucher syndrome [1,2]. The procedure of HSCT includes the ablation of the bone marrow using high-dose chemotherapy or total-body irradiation (the procedure also named conditioning), followed by the intravenous administration of the patient’s own hematopoietic stem cells (autologous HSCT) or donor-derived stem cells (allogeneic HSCT). The number of HSCTs has increased continuously worldwide by more than 7% per year, with the average number at approximately 90,000/year [3]. In total, one and a half million patients received HSCT in 2019 [3]. In recent years, a significantly improved survival of HSCT patients has been reported, which is most probably due to improvements in transplantation protocols in combination with post-transplantation supportive care strategies. The number of long-term HSCT survivors, patients who are alive without disease recurrence 12 months after HSCT, is estimated to increase five-fold by 2030 [4]. In addition, it is estimated that HSCT patients who have survived for more than 5 years without relapse have a high possibility of surviving for 15 years more [5]. Despite the significant improvements in clinical outcomes, HSCT is still associated with several severe acute and chronic complications, including relapse, graft failure, graft-versus-host disease (GvHD), infections, sinusoidal obstructive syndrome (SOS), CNS complications, hormonal disturbances, infertility, and cardiovascular toxicities [6]. These complications significantly affect the morbidity and mortality of HSCT survivors. Two-thirds of HSCT survivors develop at least one long-term complication [6]. HSCT patients with at least 5-year survival have a 4–9-fold higher mortality rate compared to the general population during the next 30-year period post-HSCT, i.e., a 30% lower life expectancy compared to the healthy population [7]. In a post-HSCT follow-up study, the incidence of late complications was reported to be 93.2% after a median follow-up time of 7.2 years (range 2–21 years) [8]. Furthermore, severe or life-threatening complications were observed in 18% of HSCT survivors [9]. Among all HSCT-related complications, cardiovascular complications are not very common (<10% for both allogeneic and autologous transplantation) but lead to a high mortality rate and significantly decrease the quality of life for long-term survivors [10]. According to a retrospective, single-center study including 265 allo-HSCT survivors, the cumulative incidence of a coronary or peripheral arterial event was 22% at 25 years post-transplantation [11]. It is, therefore, essential to reduce post-HSCT cardiovascular complications given the fact that it is a life-threatening late complication. In this review, we focus on the incidence, risk factors, mechanism, and clinical management of HSCT-related cardiovascular complications as well as the latest developments in diagnostics and treatment (as summarized in Figure 1).

## 2. Cardiovascular Complications Associated with HSCT

Acute and late cardiovascular complications are still critical limitations for patients undergoing HSCT and potential causes of morbidity and mortality in HSCT survivors [12]. The first report on HSCT-induced cardiotoxicity was in 1976 in a case study of 29 patients with leukemia, aplastic anemia, or metastatic cancer who had received ablative therapy followed by bone marrow transplantation (BMT) [13]. Two patients treated with high-dose cyclophosphamide, one of the most frequently used chemotherapy in conditioning regimens, died from fatal congestive heart failure. Autopsy investigation showed extensive myocardial necrosis, fibrin deposits and extravasation of red blood cells [13]. HSCT-related cardiovascular complications mainly include vascular abnormalities, structural heart disorders, myocardial dysfunction, and heart failure [14].

### 2.1. Short-Term Cardiotoxicity during HSCT

During HSCT/BMT or within the first 100 days post-transplantation, life-threatening cardiac complications such as arrhythmias, pericardial tamponade, acute heart failure, or cardiac arrest [15] may occur. The incidence of arrhythmia was estimated between 2–10% in adult patients [12], but lethal arrhythmia in HSCT patients sometimes occurred and was associated with longer hospitalization time, a greater probability of being admitted to an intensive care unit and a high probability of death within one year [16,17]. Myocardial infarction and severe pericardial effusion/tamponade have also been reported in HSCT recipients; however, the rate of occurrence was low [18,19,20]. The incidence of new-onset congestive heart failure (CHF) was reported to be 0.4–2.2% in HSCT recipients [21,22,23,24]. The incidence of severe acute cardiotoxicity is relatively low, ranging from 0.9–8.9%, as reported in different studies [23,25,26,27]. In a retrospective investigation including 2821 HSCT (45% allogeneic) patients (median age 22, 58% male), 26 of these patients (0.9%, 19 adults and 7 children) were either diagnosed with severe CHF, severe cardiac tamponade, significant electrocardiographic abnormalities or died due to cardiovascular dysfunction within the first 100 days post-HSCT [28].

### 2.2. Long-Term HSCT-Related Cardiovascular Complications

Several long-term cardiovascular complications, e.g., ischemic heart disease, cardiomyopathy, vascular disorder, stroke, or cardiac complications associated with comorbid conditions, such as hypertension and diabetes mellitus, have been reported [15]. Generally, there are more investigations on long-term HSCT-related cardiovascular complications compared to acute complications. In an observational study of 1244 autologous HSCT patients (1988–2002, 58% male, mean age of 44 years), Saro et al. have reported that the cumulative incidence of CHF was 4.8% at 5 years post-HSCT and increased to 9.1% at 15 years post-transplantation [29]. In this cohort, HSCT recipients had a 4.5-fold higher risk of CHF compared to the general population, while female HSCT survivors showed a higher risk of developing CHF compared to male recipients [29]. The risk factors of heart failure include age, hypertension, diabetes mellitus, and prior exposure to anthracycline treatment at doses ≥250 mg/m^2^. The authors also reported that 94% of the reported CHF cases showed a reduced ejection fraction (EF) [29]. Similarly, Armenian et al. reported in an earlier clinical study that the major risk factors of late-onset CHF (>one year post-HSCT) are anthracycline’ exposure before transplantation, number of chemotherapy cycles and comorbidities [30]. In comparison to the general population, HSCT patients showed a higher risk of developing cardiovascular diseases such as cardiomyopathy, stroke, diabetes, and ischemic heart diseases, as well as cardiovascular event-related death [31,32,33].

## 3. Factors and Causes of Cardiovascular Complications in HSCT

Several factors affect the incidence of cardiovascular complications in HSCT, including previous treatment with chemotherapy, chest radiation, chemotherapy-based conditioning, and pre-/post-HSCT comorbidities [15]. The previous treatment history of the patient is critical for the development of cardiovascular complications. Treatment regimens that include cardiotoxic drugs, i.e., anthracyclines, tyrosine kinase inhibitors (TKI) and chest radiation, subsequently increase the risk of developing both acute and late cardiovascular complications post-HSCT. In addition, chemotherapeutic agents used as a conditioning regimen for the ablation of bone marrow and prophylactic drugs used post-HSCT against GvHD and infection may contribute to both short- and long-term cardiotoxicity. Other HSCT-related comorbidities, such as acute and chronic GvHD, are also correlated with a higher risk of long-term cardiovascular complications.

### 3.1. Anthracyclines

Anthracyclines are not used in the conditioning regimen in HSCT; however, they are frequently used for the treatment of hematological malignancies, such as acute leukemia. A significantly higher risk of cardiovascular event-related death was linked to exposure to anthracyclines before HSCT [28,34]. Anthracycline-induced cardiovascular complications are well documented and characterized in clinical applications, and their toxicities are directly correlated to their cumulative dose. For instance, there is a dose-dependent correlation between pre-transplantation exposure to anthracyclines and the incidence of CHF in HSCT patients [28,29,32]. A case report from the City of Hope National Medical Center showed that HSCT survivors who received anthracycline with a cumulative dose ≥250 mg/m^2^ developed late-onset CHF (OR 3.2) [34].

One of the most commonly used anthracyclines is doxorubicin (DOX). Swain et al. reported that the risk for cardiotoxicity was 5% for DOX at a dose of 400 mg/m^2^, 26% for DOX at a dose of 550 mg/m^2^, and 48% for DOX at a dose of 700 mg/m^2^ [35]. The guidelines published by The American Society of Clinical Oncology (ASCO) define that the risk factor for cardiotoxicity is at a cumulative dose of DOX ≥ 250 mg/m^2^ or epirubicin ≥ 600 mg/m^2^ [36]. The molecular mechanism of anthracycline-related cardiotoxicity has been elucidated as increased intracellular reactive oxygen species (ROS), mitochondrial damage and dysfunction in DNA damage reparation [10,37].

### 3.2. Radiotherapy

Chest radiotherapy increases the risk of cardiomyopathy and cardiac dysfunction in HSCT patients [36,38]. An exposure dose ≥30 Gy is considered to be a risk factor for radiotherapy-induced cardiotoxicity [36]. The risk for cardiotoxicity induced by anthracycline at a lower dose might be further increased by additional low-dose radiotherapy [36]. Armenian et al. reported that coronary artery disease risk in HSCT survivors was increased by 9.5-fold in patients who received chest radiotherapy before transplantation [39]. In a group of adult survivors of childhood Hodgkin lymphoma who received radiation therapy (≥30 Gy in 48% of total patients) pre-HSCT, coronary CT angiography indicated coronary artery disease in 12 of 31 (39%) patients [40]. It is worth noting that HSCT following conditioning with radiotherapy might experience excessive iron accumulation due to red blood cell transfusion and hence cardiomyopathy via generating free radicals and reactive oxygen species (ROS) [41,42].

### 3.3. Tyrosine Kinase Inhibitors and Other Molecular-Targeted Agents

Molecular targeted therapy is becoming frequently used in cancer treatment either as a single treatment or as an integrated part of traditional chemotherapy protocols. Among different types of targeted therapy agents, tyrosine kinase inhibitors (TKIs) have been reported to be associated with cardiovascular complications. Furthermore, pre-treatment with TKIs has been reported to be associated with cardiovascular complications in HSCT survivors. TKI as post-transplantation maintenance therapy for patients with FLT3-ITD-mutated acute myeloid leukemia (AML) has been investigated in several clinical trials [43,44].

Sorafenib and midostaurin were shown to significantly reduce the relapse rate and improve overall survival in HSCT patients [10,44]; however, sorafenib was reported to be associated with adverse cardiac effects, i.e., left ventricular systolic dysfunction (LVSD) [45,46]. The Bruton TKI ibrutinib, used as a treatment for chronic lymphocytic leukemia and mantle cell lymphoma, was found to cause arrhythmia (atrial fibrillation (AF)) or hypertension in clinical patients partly due to its off-target effects [47]. Furthermore, ibrutinib was shown in multiple random studies to elevate the relative risk of AF by 4.7–5.8 -fold, and hypertension by 2.8-fold, compared to the non-treated population [48,49,50]. Moreover, a wide range of cardiac adverse effects, i.e., dysrhythmias, myocarditis/pericarditis, pulmonary hypertension, myocardial ischemia, and cerebral and peripheral vascular events, have been reported to be associated with other targeted-therapy substances [45].

### 3.4. Conditioning Regimens and Cyclophosphamide

Conditioning before transplantation is a vital part of HSCT to eliminate the bone marrow/cancer cells of the recipient, provide an immunosuppressive effect and facilitate the engraftment of transplanted donor cells. Conditioning regimens can be classified into three categories: myeloablative conditioning (MAC), reduced-intensity conditioning (RIC) or non-myeloablative conditioning. Conditioning protocols for HSCT recipients are chosen based on the patient’s age, underlying disease, and comorbidities. The conditioning regimen used can be either total body irradiation (TBI)-based or chemotherapy-based conditioning, or a combination of both. Busulfan, cytarabine, fludarabine, cyclophosphamide, idarubicin, thiotepa, and/or melphalan are the most frequently used chemotherapeutic agents in conditioning regimens [15,51].

Cyclophosphamide (Cy) is an alkylating agent used frequently in the MAC regimen at high doses for its anti-leukemia and immunosuppressive properties. It is well known that a high dose of Cy, >100 mg/kg, is correlated with cardiac damage. Cy-induced cardiac toxicity includes malignant arrhythmia, exudative pericarditis, severe myocarditis, myocardial depression, and CHF [52]. Several clinical studies showed that high-dose Cy in the conditioning regimen can cause severe and life-threatening cardiovascular complications in a dose-dependent manner. One study demonstrated the dose-dependent cardiotoxicity of Cy during the conditioning period, where 17% of the patients experienced symptoms and signs of CHF within 10 days post-Cy administration at a dose of 50 mg/kg/d for 4 days [53]. Braverman et al. followed the cardiotoxicity in HSCT recipients who were treated with Cy once (87 mg/kg) or twice daily (174 mg/kg), and found that the higher dose lead to significantly reduced cardiac function as indicated by reversible decreases in electrocardiogram (ECG) voltage and increased left ventricular mass [54]. Shinya et al. investigated Cy-induced cardiotoxicity in 811 allo-HSCT patients who received a total dose of Cy > 100 mg/kg as conditioning. The authors reported that 1.5% of these patients developed fatal cardiac failure and severe aplastic anemia at a median of 4 days after the first dose of Cy [55]. Furthermore, the incidence of cardiac failure was found to be dose-dependent and occurred at the rate of 8.5, 1.2, and 0% of the patients treated with a total dose of 200, 120, and 100 mg/kg Cy, respectively [55]. A high mortality rate (91.6%; 11/12 patients) was observed in the patients who developed fatal cardiac failure, and a total Cy dose > 200 mg/kg was recognized as the independent risk factor [55]. In a recently published study, cardiotoxicity was reported at a dose of 120 mg/kg or less in 294 patients who received a reduced total dose of Cy (100–200 mg/kg) [56]. To reduce the incidence of fatal cardiac complications and severe aplastic anemia caused by high-dose Cy, a modified conditioning regimen based on low-dose Cy and fludarabine, BuCy(low)Flu (Bu 3.2 mg/kg for 2 days; low-dose Cy 100 mg/kg; fludarabine 150 mg/m^2^), was evaluated in haploidentical HSCT [57]. Compared to the traditional BuCy regimen (Bu 3.2 mg/kg for 2 days; high-dose Cy of 200 mg/kg), the incidence of severe cardiotoxicity of BuCy(low)Flu conditioning was significantly decreased (12.80% vs. 2.17%, *p* = 0.032) without affecting the engraftment and survival [57]. Another study from our group has shown that a busulfan-cyclophosphamide (Bu-Cy) conditioning regimen in a transplantation mouse model has a significant acute impact on the vascular system. Bu-Cy-induced cardiovascular complications were observed in the smaller resistance arteries controlling blood pressure and as endothelial injury via upregulated endothelial nitric oxide synthase (eNOS) [58]. Since early endothelial dysfunction is a determining factor of atherosclerosis, further fatal cardiovascular diseases could be speculated.

Another application of Cy in HSCT is post-transplant Cy (PT-Cy) for graft-versus-host disease management. PT-Cy has become a standard prophylactic treatment for GvHD in haploidentical HSCT and is also frequently used in matched allo-HSCT [59,60,61,62]. PT-Cy recipients showed an improved 1-year overall survival and decreased mortality rate [63], and the dose of Cy used in this setting is lower than that used in the conditioning. Until now, there have been very few reports regarding the incidence and risk associated with PT-Cy-caused cardiac toxicity. Dulery et al. investigated the incidence of early cardiac toxicity in allo-HSCT patients with (*n* = 136) and without PT-Cy (*n* = 195). The results showed that exposure to PT-Cy led to a higher incidence of early cardiac toxicity within 100 days post-transplantation (19% with PT-Cy vs. 6% without PT-Cy) [64]. In another retrospective analysis of 585 allo-HSCT patients, the incidences of cardiac toxicity within 100 days post-HSCT were 6.5% for all the patients, 7.4% for PT-Cy recipients, and 5.8% for non-PT-Cy recipients [63]. Therefore, the cardiotoxicity risk caused by PT-Cy should also be taken into consideration when evaluating the cardiovascular function of HSCT survivors.

The RIC regimen was developed to be used in patients with significantly compromised organ function or underlying comorbidities in allo-HSCT [20]. The RIC regimen is recommended when patients have left ventricular ejection fraction (LVEF) ≤ 45% due to a high risk for cardiac complications with the MAC regimen [20,27]. However, cardiac complications, for example, arrhythmias, myocardial infarction, and CHF, were reported in HSCT patients who received the RIC regimen as conditioning as well [20]. The combination of melphalan and fludarabine has been reported to cause severe cardiotoxicity with an incidence of about 14% [65]. Both drugs were shown to be associated with atrial fibrillation and cardiac dysfunction [20,66].

### 3.5. Graft-Versus-Host Disease (GvHD)

Graft-versus-host disease (GvHD) is the most common life-threatening complication post-HSCT. GvHD occurs when donor immune competent T cells (the graft) recognize the recipient (the host) as a foreign body. Under this situation, the immune response activates donor T cells to gain cytotoxic capacity and then attacks the recipient to eliminate the foreign antigen(s)-bearing cells. GvHD is divided into two main clinical forms, acute and chronic GvHD [67]. GvHD is a severe post-transplantation complication and may contribute to the higher incidence of cardiac events in allogeneic HSCT patients compared to autologous HSCT patients [15,68]. There are several hypotheses regarding the effect of GvHD on allo-HSCT patients’ cardiovascular function. A retrospective clinical study showed that patients who developed grade II–IV acute GvHD also developed risk factors for cardiovascular complications, such as diabetes mellitus (DM) (RR 5.8), hypertension (HTN) (RR 9.1), and hypersensitivity lung disease (HLD) (RR 3.2) [34]. One explanation is that patients with a higher grade of GvHD are treated with steroids and calcineurin inhibitors, which subsequently cause cardiotoxicity [34]. Other than steroids and calcineurin inhibitors, ibrutinib and ruxolitinib as new treatments of GvHD are also reported to be associated with the incidence of cardiac events, such as atrial fibrillation and hyperlipidemia [69,70]. On the other hand, GvHD may directly affect artery function through induced inflammation and damage to the artery endothelium [71]. In animal models, our group showed that GvHD causes endothelial injury via increased expression of endothelial nitric oxide synthase (eNOS), which therefore affects the function of the smaller resistance arteries controlling blood pressure [58].

## 4. Diagnostics of Cardiovascular Complications

To minimize the incidence and severity of cardiotoxicities, it is generally recommended to perform a cardiac function evaluation prior to HSCT, and longitudinal cardiovascular function follow-up must be performed in HSCT survivors routinely using imaging techniques or biomarker analysis if such diagnostics are accessible. By utilizing imaging methods, cardiac dysfunction and cardiac injury can be detected earlier in HSCT patients, and therapeutic intervention may be applied in time. Before treatment, an initial screening is recommended by the ESMO (European Society for Medical Oncology) using imaging and biomarker measurement [72]. During treatment, the ASCO suggests the routine evaluation of cardiac function and frequent monitoring based on risk factor analysis for individual patients [36]. In addition, it is recommended by the American Society of Echocardiography (ASE) and the European Association of Cardiovascular Imaging (EACVI) that the cardiac function of patients who received anthracycline treatment should be evaluated before, at the end of, and 6 months after the transplantation [73]. Echocardiography examination and coronary artery function should be assessed 5- or 10-years post-transplantation based on the risk factor prediction [73]. Moreover, patients with a history of radiation exposure prior to transplantation are recommended for a yearly cardiovascular function assessment, while the involvement of a cardiologist should be considered in the case of symptomatic CHF or for those at high risk for cardiac complications.

Currently available imaging methods for cardiac function evaluation include 2D/3D echocardiography, speckle tracking (strain analysis), tissue Doppler, and cardiac magnetic resonance imaging (MRI). Echocardiography is the most widely used and well-established technique to evaluate systolic cardiac function as left ventricular ejection fraction (LVEF). An LVEF > 50% is generally accepted as the safe range to start the transplantation procedure [10]. Cardioprotective treatment and a less cardiotoxic regimen should be considered when the LVEF is less than 40% [10]. When the LVEF is more than 50% in the initial cardiac function screening but decreases by more than 10% from the initial value after treatment starts, a modification or interruption of treatment protocol should be considered during cancer treatment [10]. 3D echocardiography is highly recommended, and 2D echocardiography in combination with contrast-enhanced imaging is another option. Global longitudinal strain (GLS) analysis to quantify the longitudinal shortening provides the chance to detect the potential cardiac and left vertical dysfunction even earlier compared to basic echocardiography measurements [74,75]. Cardiac MRI is applied for cardiac function assessment when echocardiography is not available. A recent study compared cardiac MRI and echocardiography in the assessment of left ventricular function in 13 HSCT patients, and the results showed a good correlation between the two methods [76]. Although cardiac MRI may provide a more accurate measurement of minor changes in LVEF, its application is still limited by the accessibility of the instrument and the challenges in a proper acquisition setting.

Several clinical studies indicated that serum cardiac biomarkers, such as troponins and brain natriuretic peptides (BNP), may provide complementary information as a supplement evaluation for echocardiography. Furthermore, cardiac biomarkers can also be a useful tool to identify patients at risk for cardiovascular complications during HSCT. Snowden et al. measured the value of serial plasma BNP levels in 15 HSCT patients who received chemotherapy-based conditioning; significantly higher levels of BNP were observed in patients who received high-dose cyclophosphamide as part of the conditioning regimen [77]. Roziakova et al. assessed troponin T and BNP levels in allo-HSCT patients and observed persistent elevations of both markers for a period over 14 days post-HSCT in 29.7% of the patients [78]. In another study performed by the same group, it was shown that in leukemia patients treated with allo-HSCT, there was a higher level of BNP in 90.5% of the patients and a higher level of troponin T in 47.6% of the patients [79]. The reliability and accuracy of using cardiac biomarkers in cardiovascular side effect monitoring are still under investigation, and no obvious correlation between cardiac biomarker elevation and LV dysfunction was ascertained [78]. Therefore, it is not conclusive yet if biomarkers should be included as a part of routine monitoring.

## 5. Treatment and Prevention Procedures

### 5.1. Risk Prediction

There are several major risk factors contributing to HSCT-associated cardiovascular complications: (1) pre-HSCT exposure to anthracycline or chest radiation; (2) high-dose cyclophosphamide in the conditioning regimen; (3) post-transplant Cy for GvHD management; (4) other comorbid conditions, such as HTN, DM, HDL or GvHD; (5) other agents used pre-transplantation for underlying disease treatment, during transplantation/conditioning, or post-transplantation for maintenance, for example, TKIs and corticosteroids. A risk prediction model for cardiovascular disease has been developed by Armenian et al. based on the outcomes of 1828 HSCT patients who had >1 year of survival [80]. Risk scores were calculated based on selected variables: age, anthracycline dose, chest radiation, hypertension, diabetes, and smoking. A significant correlation was observed between the risk scores and the 10-year cumulative incidences of heart failure and coronary artery disease. Patients with low risk (≤3 risk factors), intermediate risk (4–5 risk factors), and high risk (≥6 risk factors) correspond to 10-year cumulative incidences of cardiovascular disease of 3.7%, 9.9%, and 26.2%, respectively [80]. Such a risk prediction model will therefore provide references to choose the optimal treatment protocol, conditioning regimens, prophylactic treatment, and necessary cardiac function monitoring for HSCT patients at different phases of transplantation. In addition, it is recommended to follow up long-term with HSCT survivors using imaging/cardiac biomarkers, for example, coronary artery calcium scoring by cardiac CT, cardiac MRI, echocardiography, lipid panel, hemoglobin A1c, and BNP every 2 years (high risk), 5 years (intermediate risk) and 10 years (low risk) [81].

### 5.2. Bone Marrow Transplant Societies’ Guideline and Recommendation

A practical guideline and recommendation for the management of long-term HSCT survivors have been published jointly by seven international bone marrow transplant societies based on retrospective studies and expert/consensus opinion [15]. In summary, it was stated that at one-year post-transplantation, a full clinical assessment for cardiovascular risk factors, such as fasting lipid panel and fasting glucose, should be performed for all HSCT survivors and annually thereafter. The risk of pre-transplantation exposure to anthracyclines/radiation should be precisely monitored and evaluated, proceeding to echocardiography screening when necessary. Other cardiovascular risk factors, for example, diabetes mellitus, hypertension and/or hyperlipidemia, should also be recognized at an early stage and managed on time.

### 5.3. European Society of Cardiology Guidelines and Recommendations

The latest “*Guidelines on Cardio-oncology*” was published in 2022 by the European Society of Cardiology (ESC) [82] in collaboration with the European Hematology Association (EHA), the European Society for Therapeutic Radiology and Oncology (ESTRO) and the International Cardio-Oncology Society (IC-OS). According to the guidelines, a comprehensive cardiovascular assessment before HSCT is recommended for all patients [82]. The recommended pre-HSCT cardiovascular evaluation includes a basic cardiovascular (CV) assessment, electrocardiogram (ECG), transthoracic echocardiography (TTE), and natriuretic peptides (NP, including brain natriuretic peptides and N-terminal pro-brain natriuretic peptides). For patients with a low risk of cardiovascular disease (CVD), only basic CV assessment and ECG are recommended at 3 months and 12 months post-HSCT and yearly afterwards. TTE and NP measurements are recommended when new cardiac symptoms occur. For patients with a high risk of CVD, the whole set of CV evaluation is recommended at 3 and 12 months post-HSCT in early surveillance. The following factors should be considered in HSCT survivors with long-term CVD: allogenic HSCT, pre-existing CVD, cancer treatment history, a high-risk conditioning regimen and GVHD.

### 5.4. Cardioprotective Agents and Treatments

There have been very limited preclinical and clinical studies performed with a focus on the preventive and therapeutic strategies for HSCT-related cardiovascular complications. The general therapeutics approach for HSCT-associated cardiac complications is chosen mainly based on the established therapeutic interventions for chemotherapy-induced cardiotoxicity, including angiotensin-converting enzyme (ACE) inhibitors (ACE-I), angiotensin II receptor blockers (ARB), and selected beta-blockers (BBs) [10,83,84]. The protective effect of ACE-I and BBs against anthracycline-induced CHF has been demonstrated in multiple clinical studies [85,86,87,88]. In non-HSCT settings, ACE-I enalapril was shown to prevent the reduction in LVEF caused by DOX and/or daunorubicin at a cumulative dose ≥200 mg/m^2^ [88]. In the HSCT setting, the effect of ACE-I and BBs as a combination treatment was investigated in 90 patients who underwent auto-HSCT in the OVERCOM trial [85]. Patients were followed for 6 months after transplantation, and no obvious LVEF reduction was detected in the enalapril and carvedilol group, as compared to a definite reduction in the placebo group [86]. Further studies are needed to investigate whether cardioprotective treatment can reduce cardiovascular complications and improve the treatment outcome in patients who received allo-HSCT.

### 5.5. Antioxidants as Cardio-Protective Treatment

Preclinical and clinical evidence has indicated that oxidative stress and ROS contribute to various cardiovascular diseases, for instance, heart failure, atherosclerosis, ischemia/reperfusion injury and cardiac arrhythmia [89]. An increased level of ROS in cardiomyocytes leads to mitochondria damage, DNA damage, protein degradation and subsequent cell death [90]. ROS overproduction was reported to contribute to cardiomyocyte dysfunction through the activation of multiple hypertrophic signaling kinases and matrix metalloproteinases, for example, tyrosine kinase, RAS and mitogen/activated protein kinase (MAPKs) [91,92,93]. In addition, oxidative stress and increased vascular superoxide production are associated with endothelial dysfunction via an ROS-induced alternation in endothelial signal transduction and redox-regulated transcription factors [94,95].

The potential application of antioxidants as a protective treatment for chemotherapy-induced cardiotoxicity has been proposed and investigated. The mechanism of the cardio-protective effect includes the activation of endogenous antioxidant molecules, the inhibition of ROS formation and the reparation of ROS-induced damage. The effect of several antioxidants has been tested in clinical studies, such as N-acetylcysteine, amifostine, L-carnitine, carvedilol, coenzyme Q10, melatonin and dexrazoxane [96]. However, most of these studies are not conclusive due to the low number of patients and lack of statistical power. Among all the antioxidants tested in clinical studies, the only one that showed a protective effect on cardiovascular toxicity is dexrazoxane. Dexrazoxane is a chelating agent which can inhibit the formation of anthracycline-iron complexes and reduce ROS levels in cardiomyocytes [97]. Dexrazoxane was shown to reduce cardiac toxicity in anthracycline-treated breast cancer patients, as indicated by less LVEF reduction in females who received anthracyclines in combination with dexrazoxane compared to the control group [98,99,100]. In children who received dexrazoxane and anthracycline for the treatment of Hodgkin’s disease and lymphoblastic leukemias, an increased risk for second malignant neoplasms and acute myeloid leukemia/myelodysplastic syndrome was observed [101,102]. Further controlled and larger multi-center trials are needed to evaluate the controversial effect of dexrazoxane in patients.

Several experimental antioxidant-based therapies have been investigated in preclinical animal models. The antioxidant probucol was shown to reduce the incidence of cardiotoxicity caused by combination treatment with doxorubicin and trastuzumab in a mouse model [103]. Left ventricular cavity dimensions and systolic parameters were preserved in mice prophylactically treated with probucol after the administration of doxorubicin and trastuzumab. Tannic acid, a PARG/PARP inhibitor and an antioxidant, was reported to reduce doxorubicin-induced cardiotoxicity and preserve its anti-cancer activity both in vitro and in vivo by preventing the activation of PARP-1 [104]. Erythropoietin treatment was shown to be potentially protective against doxorubicin-induced myocardial dysfunction, mediated by an increased number of endothelial progenitor cells [105].

Other than doxorubicin, cardiotoxicity induced by Cy is also associated with oxidative stress and nitrative stress, which cause further protein adduct formation and alternation in multiple signaling pathways. Cy and its metabolite-induced oxidative stress cause damage to endothelial cells, myocardium, and capillary blood vessels. The pathological features of Cy-induced cardiovascular toxicity include cardiomyocyte inflammation, abnormal calcium homeostasis, programmed cell death, cardiomyocyte swelling and nuclear splitting, which lead to left ventricle dysfunction and heart failure [106]. The protective effect of antioxidants on Cy-induced cardiovascular toxicity has been investigated in animal models. Several plant-derived natural antioxidants showed a promising cardio-protective effect in animal models [106]. Rats that received a single dose of Cy followed by lipoic acid treatment for 10 days showed a protective effect of lipoic acid on Cy-induced cardiotoxicity, normalized lipid peroxidation and antioxidant defenses [107,108,109]. Pre-treatment with a selenium compound in rats was shown to significantly reduce Cy-induced tissue damage and Cy-induced cardiotoxicity [110]. Another study in rats that received N-acetylcysteine pre-treatment followed by Cy treatment indicated that N-acetylcysteine attenuated Cy-induced cardiotoxicity by inhibiting oxidative and nitrosative stress and preserving the activity of antioxidant enzymes [111]. In rats, allopurinol and febuxostat as xanthine oxidase inhibitors were reported to decrease Cy-induced cardiac and bone marrow toxicities via ROS and xanthine oxidase-related pathways [112]. Nicorandil is an anti-anginal agent that functions as an adenosine triphosphate-sensitive potassium (K(ATP)) channel agonist and has nitrate-like activities, providing long-term cardio-protection [113]. Nicorandil treatment was found to eliminate Cy-induced cardiotoxicity in rats through multiple mechanisms, such as a potassium channel opening effect, stimulating endothelial nitric oxide synthase (eNOS) gene expression, and through its anti-inflammatory, antiapoptotic, and antioxidant properties [114].

Based on the current progress in elucidating the role of ROS and oxidative stress in doxorubicin/Cy-induced cardiotoxicity, further preclinical and clinical studies are needed to explore the potential application of antioxidants as cardioprotective treatment in HSCT-associated cardiovascular complications.

## 6. Future Perspective

Even though the incidence of HSCT-associated cardiovascular complications is not high, it might be life-threatening and significantly affect the quality of life of individuals, especially when other post-transplantation complications present as well. Causes, risk factors and potential mechanisms of cardiotoxicity in stem cell transplantation have been discussed in the present review. Most of the published clinical studies are based on retrospective analyses of adult HSCT patients. More cohort clinical trials that include both adult and pediatric patients are urgently warranted to systematically analyze all the risk factors for both long-term cardiac complications and short-term toxicity to be able to establish a more concrete treatment strategy and management guideline specific to HSCT-related cardiac adverse effects.

Due to the insufficient clinical and preclinical investigations and results, cardioprotective treatments used in HSCT are mainly based on the interventions developed for chemotherapy-induced cardiotoxicity. More preclinical and clinical data are necessary to improve the transplantation protocols, imaging/blood biomarkers, and preventative/curative treatments specific to transplantation-associated cardiac events. This could reduce the incidence of cardiac complications, which, in turn, can enhance clinical outcomes and improve quality of life for HSCT patients.

## Data Availability

Not applicable.
